# Quality of maternal and newborn health care at private hospitals in Iraq: a cross-sectional study

**DOI:** 10.1186/s12884-023-05678-3

**Published:** 2023-05-09

**Authors:** Hannah Tappis, Rebaz Lak, Riyadh Alhilfi, Aso Hameed Zangana, Falah Wadi, David Hipgrave, Shaimaa Ibrahim

**Affiliations:** 1 Department of International Health, Johns Hopkins Center for Humanitarian Health, Johns Hopkins Bloomberg School of Public Health, Baltimore, USA; 2 Kurdistan Higher Council of Medical Specialties, Erbil, Iraq; 3 Ministry of Health, Baghdad, Iraq; 4 Kurdistan Ministry of Health, Erbil, Iraq; 5 UNICEF, Erbil, Iraq; 6 UNICEF Iraq Country Office, Baghdad, Iraq

**Keywords:** Iraq, Private sector, Maternal health, Newborn health, Cesarean birth, Quality of care

## Abstract

**Background:**

Approximately 25% of facility births take place in private health facilities. Recent national studies of maternal and newborn health (MNH) service availability and quality have focused solely on the status of public sector facilities, leaving a striking gap in information on the quality of maternal and newborn care services.

**Methods:**

A rapid cross-sectional assessment was conducted in November 2022 to assess the quality of MNH services at private hospitals in Iraq. Multi-stage sampling was used to select 15% of the country’s 164 private hospitals. Assessment tools included a facility assessment checklist, a structured health worker interview tool, and a structured client exit interview tool. Data collection was conducted using KoboToolbox software on Android tablets, and analysis conducted using SPSS v28.

**Results:**

All hospitals visited provided MNH services and had skilled personnel present or on-call 24 h/day, 7 days/week. Most births (88%) documented between January and June 2022 were cesarean births. Findings indicate that nearly all hospitals have the human resources, equipment, medicines and supplies necessary for quality antenatal, intrapartum and early essential newborn care, and many are also equipped with special units and resources needed to care for small and sick babies. However, while resources are in place for basic and advanced care, there are gaps in knowledge and practice of high-impact interventions that require few or no resources to perform, including skin-to-skin thermal care and support for early initiation of breastfeeding. Person-centered maternity care scores suggest that private hospitals offer a positive experience of care for all clients, however there is room for improvement in provider–client communication.

**Conclusions:**

This assessment highlights the need for deeper dives into factors that underly decisions about how and where to give birth, and both understanding and practice of early essential newborn care and pre-discharge examinations and counseling at private healthcare facilities in Iraq. Engaging private health facility staff in efforts to monitor and improve the quality of maternal and newborn care, with a focus on early essential newborn care and provider–client communication for all clients, will ensure that women and newborns benefit from the best care possible with available resources.

## Background

The Ministry of Health of Iraq recognizes that the health of women and children is key to progress on all development goals, and has committed to improving equity of access to quality maternal and newborn care services in an effort to reach global targets for maternal and neonatal mortality and stillbirth reduction by 2030 [1, 2]. The majority of births (84%) take place in health facilities, with the private sector playing an important role in delivering services across the continuum of care from pregnancy to childbirth and the postpartum and postnatal period [3]. Little is known, however, about the quality of care provided in the private sector.

The *Lancet Global Health Commission on High Quality Health Systems,* which called for a better understanding of the dimension of quality for health systems in resource-limited settings, lays out compelling evidence that merely accessing or reaching the doorstep of a healthcare system does not equate to or ensure receipt of high-quality care [4]. Similarly, a growing body of maternal and newborn research has documented high variation in quality of care available within and across countries, including evidence that many women are mistreated during childbirth and that newborn babies are often neglected during the first hours of life [5, 6]. A recent modeling exercise suggests that improving quality of maternal and newborn health care (with no changes in current levels of access or utilization) in low- and-middle-income countries could prevent approximately one-quarter of maternal deaths, neonatal deaths and stillbirths [7]. However the potential benefits of improving quality of care are likely even greater, given the impact that perceptions of quality and experiences of care have on decisions if, when, and where to seek care [8, 9].

Several countries, including Iraq (an upper-middle-income country in the Middle East), have seen a rapidly expanding private sector involved in the delivery and acceptance of basic health services over the past decade [10, 11]. A study of 70 countries revealed that the private health care industry is accountable for the delivery of more than a third of the world's maternal health care [12]. In low- and middle-income countries, the private health sector holds a mean market share of 44% among those who use prenatal care and a mean market share of 40% among those who use delivery care [13]. Stakeholders in maternal and newborn health are increasingly recognizing that in mixed health systems, engagement of both public and private sectors in the delivery of quality services for maternal, newborn and child care will be critical to achieving the Sustainable Development Goals (SDGs) [14]. Yet numerous barriers to effective private sector engagement have been identified, including ineffective governance, inadequate information, and a lack of experience in how the public and private sectors may collaborate to achieve national health goals like universal health coverage and reduction of preventable maternal and newborn mortality and morbidity [15].

In Iraq, there are 164 private hospitals distributed throughout the country; roughly two thirds (106) are located in the capital city of Baghdad and the Kurdistan Region of Iraq (KRI) and the remaining third across the other 15 governorates in the country [16]. Data reported to the Ministry of Health in 2021 suggests that approximately 25% of facility births take place in private facilities, and that as high as 85% of births at private facilities are cesarean births [16]. Caesarean birth is one of the most common surgeries performed worldwide, and is a lifesaving intervention for women and babies when conducted safely, timely, and for the right reason [17]. However, when a cesarean is conducted unsafely or without a medical indication, it can increase risk of adverse maternal and perinatal outcomes, add to both health system costs and out-of-pocket expenses, and may jeopardize progress towards the SDGs for maternal and newborn health [18].

While the Ministry of Health’s private sector department must authorize private hospitals before they may set up maternal and newborn health units and services, there is no routinized audit or oversight of the quality of care offered in private hospitals and no uniform criteria for monitoring this. Currently private hospitals are only required to report to the Ministry of Health on the number of births occurring on site each month, disaggregated by spontaneous vaginal births and cesarean births. In 2021, the Ministry of Health reported that cesareans account for 35% of births in public facilities and over 80% in private facilities [16]. There are no other formal mechanisms for public–private sector collaboration or regulation of private hospitals and clinics with regards to uniform standards of care or reporting of health outcomes. Recent national studies of maternal and newborn health service availability and quality have focused solely on the status of public sector facilities, leaving a striking gap in information on the quality of maternal and newborn care services received by approximately one-fifth of childbearing women.

In order to understand the contribution of the private sector to universal health coverage and progress towards targets for maternal and newborn mortality reduction in Iraq, a better understanding of the private sector’s involvement in delivering maternal and newborn health care is needed.

## Methods

A rapid cross-sectional assessment was conducted in November 2022 to assess the quality of maternal and newborn health services at private hospitals in Iraq. Assessment tools included a health facility inventory and document review checklist (Tool 1), a structured health worker interview tool (Tool 2), and a structured client exit interview tool (Tool 3). Tools were adapted from the *Demographic and Health Surveys (DHS) Program Service Provision Assessment* which was revised in 2022 to better capture structures and processes required for quality maternal and newborn health service provision (https://www.dhsprogram.com/Methodology/Survey-Types/SPA.cfm). Where content on priority topics in Iraq’s *National Reproductive, Maternal, Neonatal, Child and Adolescent Health Strategy* were lacking, namely management of select obstetric complications and small and sick newborn care, additional content was added from the *Averting Maternal Death and Disability EmONC Assessment Toolkit (2015)* (https://www.publichealth.columbia.edu/research/averting-maternal-death-and-disability-amdd/toolkit) and *NEST360/UNICEF Health Facility Assessment Toolkit (2021*) (https://nest360.org/project/hfa/).

Assuming a minimum of 85% of health facilities meet national maternal and newborn health quality standards, a sample of 15% (*n* = 24) of the private hospitals in the country was determined to be sufficient to allow the assessment team to measure indicators of interest (such as the proportion of facilities with equipment and supplies necessary for routine intrapartum and newborn care) with ± 15% precision, 95% confidence and 80% power. A multi-stage sampling strategy was used to select hospitals for participation in this assessment. First, governorates in Iraq were stratified into three regions: north (KRI), central and south. Next, two to three governorates in each region were purposively selected in consultation with representatives of the Ministry of Health of Iraq and Ministry of Health in KRI. Selected governorates included Erbil and Sulaymaniyah (KRI), Baghdad, Karbala, and Salahaddin (central), and Thi-Qar and Basra (south). Finally, a sample proportional to the distribution of hospitals across these governorates was randomly selected using SPSS v28.

Data collection teams, each consisting of two physicians trained on assessment protocols and tools, visited selected health facilities in November 2022. UNICEF and Ministry of Health co-investigators conducted a three-day training on assessment objectives, methods and tools for all data collectors and supervisors in early November 2022. Site visits were two days in duration, and coordinated by supervisors based in the governorate’s Directorate of Health between 14 – 21 November. At each facility, teams were expected to complete the health facility inventory and document review checklist (Tool 1), interview up to four health workers responsible for maternal and newborn care (Tool 2), and interview up to four women being discharged after childbirth. Teams were instructed that health worker interviews should include a mix of doctors, midwives and nurses (depending on staffing at the facility), and where possible postpartum exit interviews should include a mix of women with spontaneous vaginal delivery and cesarean birth.

All data collection was conducted using KoboToolbox software on Android tablets. Interviews were primarily conducted in Arabic at facilities in central and southern Iraq, and in Kurdish in KRI. Following completion of all assessments, data were exported from KoboToolbox and analyzed using SPSS v28. Analysis included descriptive statistics, and calculation of person-centered maternity care score using a 13-item scale developed by Afulani et al. to measure experience of care [19].

The assessment protocol was reviewed and endorsed by the Ministry of Health of Iraq and Ministry of Health in the Kurdistan Region of Iraq, and approved by the Kurdistan Higher Council of Medical Specialties Research Protocol Ethics Committee (meeting code 1832, 13 October 2022).

## Results

All 24 selected private hospitals agreed to participate in the assessment, resulting in a total of 24 health facility inventory and record review checklists completed, 92 interviews with skilled health personnel responsible for intrapartum care, and 93 postpartum client exit interviews including 22 (24%) with spontaneous vaginal births and 71 (76%) with cesarean births.

All private hospitals visited provided maternal and newborn health services and had skilled health personnel present at the facility at all times or officially on call 24 h/day, 7 days/week in case of emergencies. Approximately half of the hospitals visited (54%, *n* = 13) allow birth companions to be present during labor and delivery for vaginal births. Of the 24 private hospitals visited, 96% (*n* = 23) perform both spontaneous vaginal births and elective cesarean births. Only one facility reported only performing elective cesareans. The vast majority (88%) of births documented between January and June 2022 were cesarean sections. During this period, these hospitals recorded an average of 23 spontaneous vaginal births per month (range 0–155) and 152 cesarean births (range 10–818) per month.

On average, hospitals had two delivery rooms and six operating theaters. All supporting spontaneous vaginal births had a newborn corner in the delivery room, and all 24 hospitals had special units for care of small and sick newborns. Only 42% (*n* = 10) reported keeping mothers and babies together (rooming-in) after birth, which is a WHO-recommended evidence-based practice for all mother/baby dyads without complications, including after cesarean births.

### Availability of medicines, supplies and equipment

All facilities had nearly all medicines, supplies and equipment necessary for routine intrapartum and newborn care, as well as for care of small and sick newborns typically secondary (level 2/special care) facilities. However, only 71% (*n* = 17) had a fetal stethoscope for monitoring of fetal well-being (Table 1). Although all selected facilities had newborn sized ambu bags and masks for resuscitation of babies not breathing at birth, not all had size zero masks readily available. Supplies for small and sick newborns with trouble feeding were also lacking in some facilities, and while most had CPAP machines and phototherapy equipment, fewer had medicines for seizure management.

**Table 1:** Availability of medicines, supplies and equipment for intrapartum and newborn care (*n* = 24 facilities)

**Routine intrapartum and newborn care**			
**Availability in or near the delivery room**		**Availability in or near the operating theater**	
***Item***	***% (n)***	***Item***	***% (n)***
Digital or manual BP apparatus	96% (23)	Operating table and light	100% (24)
Stethoscope	88% (21)	Anesthesia machine	100% (24)
Sonic aid (Fetal stethoscope/ pinard)	71% (17)^a^	Uninterrupted oxygen source with regulator and pulse oximeter	100% (24)
Cardiotocography (CTG)	71% (17)	Ketamine	100% (24)
Oxytocin	96% (23)	Oxytocin	100% (24)
Misoprostol	88% (21)	Misoprostol	96% (23)
Newborn size self-inflating bag	92% (22)	Newborn size self-inflating bag	96% (23)
Newborn mask size 0	75% (18)	Newborn mask size 0	83% (20)
Newborn mask size 1	92% (22)	Newborn mask size 1	100% (24)
Newborn scale	92% (22)		
Vitamin K	92% (22)		
**Care for small and sick newborns**			
*Level 2*^*b*^			
Sterile feeding syringes		79% (19)	
Measurement and feeding cups		63% (15)	
Breast pump		58% (14)	
Radiant warmer and accessories		92% (22)	
Incubator		96% (23)	
Infusion pump with newborn-sized accessories		88% (21)	
Uninterrupted oxygen supply with regulator		96% (23)	
CPAP and accessories		92% (22)	
Phototherapy equipment and accessories		96% (23)	
Diazepam		75% (18)	
Epinephrine		79% (19)	
Phenobarbital sodium		88% (21)	
Injectable antibiotic (e.g., ceftriaxone, benzylpenicillin, gentamicin)		92% (22)	
Caffeine citrate		79% (19)	
Glucose hyper		96% (23)	
Corticosteroid (dexamethasone, betamethasone)		92% (22)	
*Level 3*^*b*^			
Mechanical ventilation equipment & accessories		54% (13)	
Surfactant doses		25% (6)	

^a^Availability of Sonicaid was only assessed at 20 of 24 selected hospitals reporting provision of antenatal care

^b^Requirements for inpatient care for small and sick newborns at different health system levels adapted from WHO & UNICEF. Survive and thrive: transforming care for every small and sick newborn. Geneva: World Health Organization; 2019

### Health worker knowledge of evidence-based intrapartum and newborn care practices

When asked to list out steps in essential care for every mother and newborn, or issues to check in pre-discharge examinations, a notable proportion of health workers missed critical elements of evidence-based care (Table 2). For example, only 62% mentioned that a uterotonic should be administered within 1 min after birth to prevent postpartum hemorrhage. Only 75% mentioned skin-to-skin contact and only 50% mentioned support to initiate breastfeeding as components of essential newborn care. Fewer than 50% of health workers mentioned that mothers should be checked for breathing difficulties, signs of anemia or malnutrition, breast engorgement, deep vein thrombosis or depression. Similarly, less than half of respondents mentioned that newborns should be checked for signs of fever or infection, umbilical cord infection, or eye swelling or discharge prior to discharge.

**Table 2:** Health worker knowledge of evidence-based intrapartum and newborn care practices (*n* = 92 skilled health personnel)

**Health worker characteristics**	
Years working in current position [Mean(SD)]	6 (8)
Currently working in more than one health facility	96% (92)
Currently working in both public and private health facilities	51% (49)
Received refresher or in-service training on maternal and newborn health in the last 2 years	69% (66)
**Health worker knowledge**	
*Active management of the third stage of labor*	
Administer uterotonic within 1 min after birth	62% (57)
Check uterine tone and massage if soft	75% (69)
*Essential newborn care*	
Drying of baby	79% (73)
Ensure baby is breathing/crying	71% (65)
Provide thermal protection (skin to skin)	75% (69)
Ensure mother initiates breastfeeding	50% (46)
Assess/examine newborn within 1 h	42% (39)
Weigh newborn	57% (52)
Cut cord with sterile blade / scissors	64% (59)
Vitamin K injection	49% (45)
*Newborn resuscitation*	
Call for help	91% (84)
Explain to mother the condition of baby	19% (17)
Place newborn face up	27% (25)
Wrap or cover baby	40% (37)
Position baby’s head so neck is slightly extended	21% (19)
Suction mouth and nose if airway blocked	83% (76)
Start ventilation using bag and mask	83% (76)
*Care for low birthweight newborns*	
Ensure that the baby is warm with skin-to-skin with mother (kangaroo technique)	46% (42)
Ensure baby is warm by placing baby in incubator/radiant warmer	74% (68)
Provide extra support to establish breastfeeding	44% (40)
Monitor ability to breastfeed	59% (54)
Assess for jaundice	37% (34)
Assess need for oxygen supplementation	65% (60)
Monitor baby for the first 24 h	46% (42)
*Examination of newborn prior to discharge*	
Baby breastfeeding well	87% (80)
Proper positioning for breastfeeding	55% (51)
Color tone of baby	82% (75)
Signs of infection (fever)	35% (32)
Difficulty breathing	87% (80)
Eye swelling or discharge	13% (12)
Umbilical cord	45% (41)
Baby’s weight	41% (38)
Alertness of baby	59% (54)
*Examination of mother prior to discharge*	
Vaginal bleeding	99% (91)
Signs of infection (fever)	50% (46)
Blood pressure	90% (83)
Abdominal tenderness	65% (60)
Size and firmness of uterus	75% (69)
Deep vein thrombosis	17% (16)
Breast engorgement	25% (23)
Signs of anemia	49% (45)
Lochia (vaginal discharge)	68% (63)
Signs of depression	9% (8)
Dribbling urine	71% (65)
Cough or breathing difficulties	31% (29)
Signs of malnutrition or food insecurity	25% (23)

### Experience of care

Person-centered maternity care scores suggest that private hospitals offer a positive experience of care for all clients, regardless of the mode of delivery (Table 3). Nearly all exit interview participants (including the few who reported experiencing mistreatment during their stay) also reported that they would recommend the facility to family and friends, indicating a high degree of satisfaction with services. Nevertheless, health worker and client interviews also indicate that provider–client communication may be an area in need of improvement. Although nearly all women participating in exit interviews reported feeling they could ask health workers any questions they had, only 19% of health workers mentioned informing the mother what was happening as a step of resuscitation of babies not breathing at birth (Table 2), and fewer than 60% of clients interviewed received explanations of medicines provided or felt involved in decisions about their care (Table 3). Exit interviews also revealed potential gaps in postpartum counseling practices. For example, less than a third of women interviewed were counseled on the risks of using bottles and pacifiers, and less than two-thirds on exclusive breastfeeding or childhood vaccination schedules.

**Table 3:** Characteristics, experiences and perceptions of care reported by postpartum exit interview participants

	Clients with a spontaneous vaginal birth (*n* = 22)	Clients with a cesarean birth (*n* = 71)	**Total (*n* = 93)**
**Client characteristics**			
Age [Mean (SD)]	28 (5)	28 (7)	**28 (7)**
Highest level of school attended			
Primary school	36% (8)	30% (21)	**31.1% (29)**
Secondary school	18% (4)	25% (18)	**23.7% (22)**
Higher education	46% (10)	42% (30)	**43% (40)**
Declined to answer	–-	3% (2)	**2% (2)**
Parity [Mean (SD)]	1.59 (1.56)	1.56 (1.64)	**1.57 (1.62)**
Past cesarean births [Mean (SD)]	0.12 (0.33)	1.35 (1.09)	**1.03 (1.09)**
Previous births with same facility or health worker [Mean (SD)]	0.76 (1.30)	0.71 (0.96)	**0.73 (1.05)**
**Person-centered maternity care score**			
Did the doctors, midwives or other healthcare providers call you by your name?	82% (18)	77% (52)	**78% (70)**
Did the doctors, midwives or other healthcare providers treat you with respect?	100% (22)	99% (67)	**99% (89)**
Did the doctors, midwives or other healthcare providers treat you in a friendly manner?	100% (22)	100% (68)	**100% (90)**
During examinations in the labor room, were you covered up with a cloth or blanket?	82% (18)	–-^a^	**20% (18)**
Did you the feel the health workers involved you in decisions about your care?	59% (13)	75% (51)	**71% (64)**
Did the health workers ask your permission before doing examinations and procedures on you?	82% (18)	90% (61)	**88% (79)**
During delivery (labor), did you feel like you were able to be in the position of your choice (lithotomy, squatting, etc.)?	18% (4)	–-^a^	**4% (4)**
Did the health workers explain to you why there were carrying our exams or procedures?	68% (15)	87% (59)	**82% (74)**
Did the health workers explain to you why and how they were giving you any medicines?	55% (12)	93% (63)	**83% (75)**
Did you feel like you could ask the health workers any questions you had?	96% (21)	100% (68)	**99% (89)**
Did the health workers talk to you about how you were feeling?	73% (16)	88% (60)	**84% (76)**
When you needed help, did you feel the health workers paid attention?	96% (21)	100% (68)	**99% (89)**
Did you feel the health workers took the best care of you?	100% (22)	100% (68)	**100% (90)**
Person-Centered Maternity Care Score (13-item scale, with 0–3 points possible for each item: 0 = no, never, 1 = yes, a few times, 2 = yes, most of the time, 3 = yes, all of the time) [Mean (SD)]	28.4 (6.4)	28.6 (4.1)	**28.5 (4.8)**
Minimum	14 of 39	16 of 33^a^	**14**
Maximum	38 of 39	33 of 33^a^	**38**
**Client recall of counseling provided on postnatal care**			
Using family planning after the birth of your baby to prevent unwanted pregnancy or space your next birth			**24% (15)**
Exclusive breastfeeding (not giving your baby any fluids or food in addition to breastmilk)			**68% (43)**
Where to access breastfeeding support in the community			**25% (16)**
Signs that the baby is hungry			**30% (19)**
Risks of using feeding bottles, teats and pacifiers			**32% (20)**
Nutrition (what is good for you to be eating after having your baby)			**71% (45)**
The importance of taking iron tablets after having your baby			**49% (31)**
What to do if you feel sad or depressed after giving birth			**6% (4)**
Health signs and symptoms for which you must immediately come back to the facility			**57% (36)**
Health signs and symptoms for which you must immediately bring your baby back to facility			**54% (34)**
Registration of the birth of your baby			**60% (38)**
Vaccinating, complete your vaccination and vaccinate your baby			**57% (36)**
How to engage and play with your baby			**10% (6)**
When to next visit a health facility for health checks for you and the baby			**70% (44)**
**Client perceptions of service quality**			
Felt the amount of time waiting to see a health worker was a problem			**5% (5)**
Felt the facility environment was clean			**95% (88)**
Felt they were physically mistreated (treated roughly, pushed, slapped, squeezed, pinched, restrained, etc.)			**3 (3%)**
Felt they were verbally mistreated (treated rudely, shouted at, insulted, etc.)			**2 (2%)**
Would recommend facility to family or friends			**99% (92)**

^a^Questions related to examinations and position of choice during labor and birth were not asked in exit interviews with postpartum women who gave birth via cesarean

### Referral

Approximately two-thirds of hospitals visited report receiving patients referred in from other facilities, and also referring out patients with obstetric and/or newborn complications. Of the 24 facilities visited, only 38% (*n* = 9) have a designated focal person for managing referral communications and only 67% (*n* = 16) have emergency transport available.

### Documentation

**Fig. 1:**
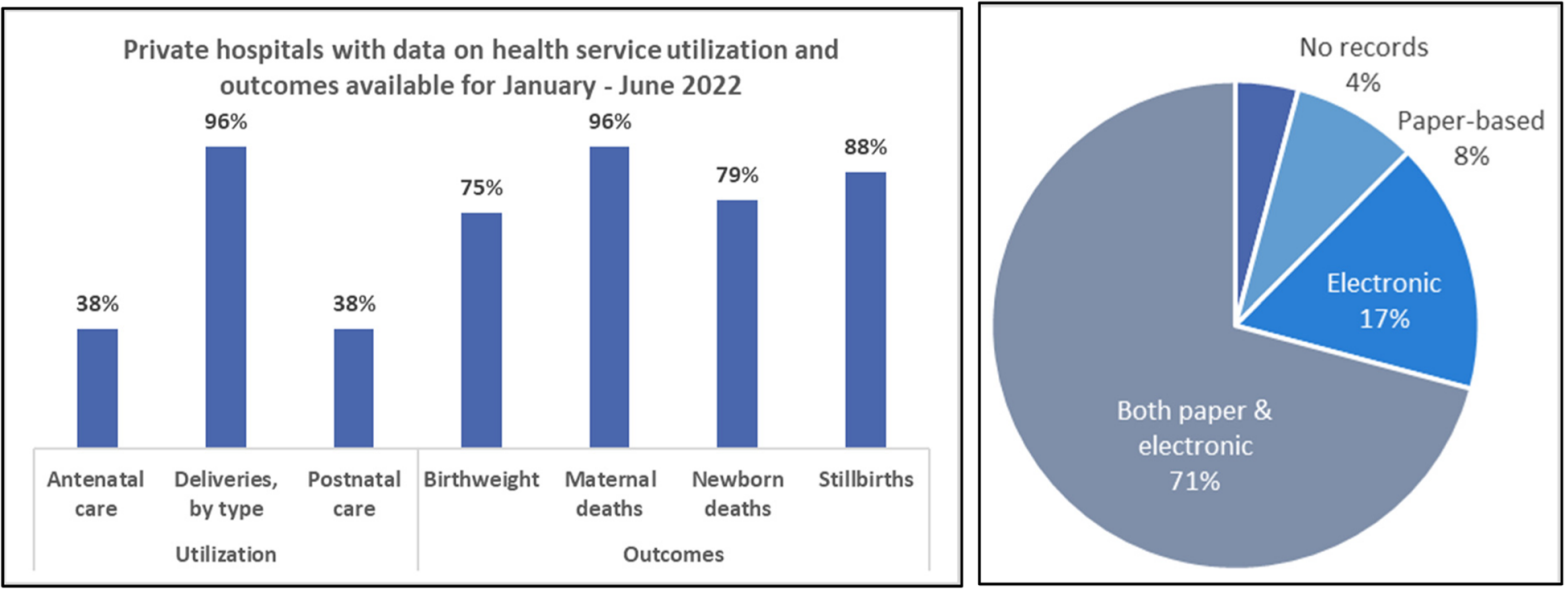
Percent of hospitals with data available on maternal and newborn health services, and type of record available

The majority of private hospitals visited used a combination of paper charts and electronic medical records. Nearly four-fifths (79%, *n* = 19) indicated that they have standard operating procedures for reporting maternal deaths, and approximately two-thirds (67%, *n* = 16) indicated that they have procedures for reporting perinatal or newborn deaths. Although nearly all facilities (96%, *n* = 23) were able to produce data on the number of births in the first six months on 2022, fewer (88%, *n* = 21) stated that they regularly compile reports on maternal and newborn health services. Only 42% of facilities (*n* = 10) reported having a functional mechanism for recording and sharing outcomes of cases referred in and out. The number of facilities able to share data on other key maternal and newborn health indicators varied, as shown below (Fig. 1).

## Discussion

This rapid assessment provides a snapshot of the quality of maternal and newborn care services at a representative sample of private hospitals in Iraq in late 2022. Overall, findings indicate that nearly all private hospitals have the human resources, equipment, medicines and supplies necessary for quality intrapartum care and early essential newborn care, and many are also equipped with special units and resources needed to care for small and sick babies. However, while resources are in place for basic and advanced care, there are apparent gaps in knowledge and practice of high-impact interventions that require little to no resources to perform, including skin-to-skin thermal care and support for early initiation of breastfeeding. Considering nearly all health workers interviewed (91%, *n* = 92) work in multiple facilities and approximately half (51%, *n* = 49) are dual practitioners working in both public and private sector facilities, it is fair to assume that investments in quality improvement are needed across the entire health system. As a rapid assessment conducted to provide the Ministry of Health with initial insights on quality of health services at private hospitals with limited government oversight, this study has a number of limitations. First, the sample size was limited to the most precise estimate feasible within practical resource and time constraints; a larger sample of health facilities would have allowed for more precise estimates of resource availability. Sample size and power calculations were used to ensure a representative sample of health facilities, not necessarily of health workers or clients giving birth at facilities. A more robust study powered to provide a statistically representative assessment of health worker knowledge and behaviors, as well as of client experiences of care, would provide greater certainty in identification of areas of concern. Additionally, Afulani et al.’s person-centered maternity scale incorporated in *Demographic and Health Surveys (DHS) Program Service Provision Assessment* was created and validated for interviews of women with vaginal births; it has not yet been validated for cesarean births where there may be additional factors to consider. Finally, there are also risks of response or courtesy bias that should be considered in interpretation of results. Data collectors were identified as being affiliated with the Ministry of Health, which may have biased participant responses. Client exit interviews were conducted on site around the time of discharge, which also may have biased responses if participants felt information provided could affect care for themselves or their families. These limitations notwithstanding, this study is an important first step in closing the gap in information on quality of maternal and newborn care in Iraq, particularly in the context of a continually expanding private healthcare sector and rising cesarean rates.

Concerns about quality of care and maternal and newborn outcomes, including breastfeeding initiation and support, in settings with rising cesarean birth rates are not unique to Iraq [20, 21]. Cesarean rates in the Middle East are among the highest in the world [22]. The rising global caesarean rate, which increased from 7 to 21% between 1990–2020 is largely driven by non-medically indicated caesareans and can be attributed to a complex web of factors, including women’s and families’ preferences, health workers’ beliefs and preferences, convenience, remuneration, healthcare organization and financing structures [16, 19, 23, 24] Some of these factors are country-specific, but others are universal and aligned with values and perceptions associated with contemporary culture across nations.

In this context, reducing the practice of non-medically indicated cesareans has proven challenging, but there is substantial evidence that introduction of action-oriented monitoring strategies and systems to evaluate trends in cesarean rates, care practices and outcomes have identified opportunities to improve quality of care for mothers and newborns [25, 26]. Other countries in the region have documented an increase in neonatal intensive care unit admissions associated with an increase in cesarean rates, including scheduling of elective procedures at late preterm and early term gestation, and instituted practices to reduce the frequency of pre-term births [27]. Quality improvement initiatives in other regions have also shown that increased attention to early essential newborn care is associated with improved outcomes following cesarean births [28].

Although research on drivers of cesarean rates and quality of care for mothers and newborns after cesarean births in Iraq is relatively scarce, concerns about the potential contribution of the expansion of the private healthcare sector to increasing cesarean rates and impacts on breastfeeding rates and health outcomes have been documented for over a decade [29, 30]. This rapid assessment highlights the need for deeper dives into factors that underly decisions about where to give birth, mode of delivery, and both understanding and practice of early essential newborn care (including early initiation of breastfeeding) and pre-discharge examinations and counseling at public and private healthcare facilities in Iraq. At the same time, improving attention to maternal mental health and quality of early essential newborn care and counseling prior to discharge from health facilities after childbirth does not need to wait for further research. This assessment identified a number of gaps that could be addressed through refresher training, site-level quality improvement initiatives and closer monitoring of care processes. Establishment of a policy framework to promote public–private partnership for maternal and newborn health could provide the foundation for accreditation of private hospitals, formalization of referral processes, and sharing of a key set of maternal and newborn health service coverage and quality indicators to monitor progress towards universal health coverage and quality care.

## Conclusions

Cooperation of national and subnational Ministry of Health authorities as well as all selected private hospitals in this assessment is a signal of both government and private sector interests in collaborating to ensure women and children receive quality care. Establishing a policy framework to promote public–private partnership and engaging private health facility staff in efforts to monitor and improve the quality of maternal and newborn care, with a focus on early essential newborn care and provider–client communication for all clients, will ensure that women and newborns benefit from the best care possible with available resources.

## Data Availability

Assessment datasets are available from the corresponding author on reasonable request and signature of a data use agreement with UNICEF Iraq. Requests should be submitted to baghdad@unicef.org.

## Abbreviations

CPAP: Continuous positive airway pressure
CTG: Cardiotocography
KRI: Kurdistan Region of Iraq
MNH: Maternal and newborn health
SD: Standard deviation
SDGs: Sustainable Development Goals
